# Hypercapnia promotes maladaptive airway and vascular remodeling in mice

**DOI:** 10.1172/JCI196928

**Published:** 2025-08-26

**Authors:** Masahiko Shigemura, Felix L. Nuñez Santana, S. Marina Casalino-Matsuda, David Kirchenbuechler, Radmila Nafikova, Fei Chen, Zhan Yu, Yuliana V. Sokolenko, Estefani Diaz, Suchitra Swaminathan, Suror Mohsin, Rizaldy P. Scott, Lynn C. Welch, Chitaru Kurihara, Emilia Lecuona, G.R. Scott Budinger, Peter H.S. Sporn, Jacob I. Sznajder, Ankit Bharat

**Affiliations:** 1 Division of Thoracic Surgery,; 2 Division of Pulmonary and Critical Care Medicine,; 3 Center for Advanced Microscopy,; 4 Robert H. Lurie Comprehensive Cancer Research Center, and; 5 Department of Pathology, Feinberg School of Medicine, Northwestern University, Chicago, Illinois, USA.; 6 Research Service, Jesse Brown Veterans Affairs Medical Center, Chicago, Illinois, USA.

**Keywords:** Muscle biology, Pulmonology, COPD, Extracellular matrix, Structural biology

## Abstract

CO_2_ isn’t just a gas—it can remodel lungs and worsen COPD, but may be reversible with CO_2_-lowering therapies.

**To the Editor:** Chronic obstructive pulmonary disease (COPD) is a leading cause of morbidity and mortality worldwide, characterized by persistent airflow obstruction due to structural alterations in the lungs. Acute exacerbations and disease progression are frequently associated with hypercapnia (elevated arterial carbon dioxide [CO_2_]), which correlates with worse clinical outcomes. Although traditionally considered a consequence of disease progression, hypercapnia has recently emerged as a potential driver of COPD pathogenesis.

We previously demonstrated that elevated CO_2_, independently of hypoxia and acidosis, acts as a gaso-signaling molecule that increases airway smooth muscle cell (ASMC) contractility in murine and human systems, thereby promoting airway hyperreactivity and constriction (1). In patients with COPD, chronic hypercapnia is associated with increased airway resistance, which can improve with noninvasive ventilation aimed at correcting hypercapnia (1). However, the structural consequences of hypercapnia in the lungs, particularly its effect on airway architecture, extracellular matrix (ECM) organization, and mesenchymal cell function, remain unclear.

We hypothesized that (a) chronic hypercapnia induces lung remodeling by altering the resident mesenchymal cell phenotype and that (b) these changes, while potentially reversible, contribute to COPD pathology. To test this, we exposed C57BL/6J mice to normoxic hypercapnia (10% CO_2_, 21% O_2_) or room air for up to 21 days (Figure 1A) (1). H&E staining showed no parenchymal destruction or inflammation in hypercapnia-exposed lungs but identified abnormalities in peribronchial and perivascular regions (Figure 1B). Immunostaining for α–smooth muscle actin (α-SMA) showed increased smooth muscle mass in bronchioles (peak: day 7) and pulmonary arteries (peak: day 21) (Figure 1C). In vivo and in vitro analyses showed no increase in lung SMC proliferation (Supplemental Figure 1, A and B), suggesting hypertrophic rather than hyperplastic remodeling. Masson’s trichrome staining revealed substantial ECM deposition (peak: day 21) in the bronchovascular sheath (Figure 1C). These changes were partially reversible: 14 days of recovery in room air markedly reduced smooth muscle thickening and ECM deposition (Figure 1C). CO_2_-driven changes may be duration- and dynamics-dependent.

In vitro, ASMC and pulmonary arterial SMCs (PASMCs) cultured under high CO_2_ (~120 mmHg, pH 7.4) for 3 days increased α-SMA and F-actin, consistent with a contractile hypertrophic phenotype (Supplemental Figure 2, A–D). *COL1A1* was unchanged (Supplemental Figure 1C). In contrast, lung fibroblasts exposed to high CO_2_ upregulated α-SMA and *Col1a1* (Supplemental Figure 2, E and F), suggesting myofibroblast differentiation. Transcriptomics profiling identified *Ltbp2*, a marker of myofibroblasts (2), as a hypercapnia-responsive ECM gene in fibroblasts (Supplemental Figure 2, G and H), but not in SMCs (Supplemental Figure 1C). These findings suggest that hypercapnia induced phenotypic shifts toward a contractile smooth muscle– and myofibroblast-like phenotype.

To evaluate the translational relevance, we cultured precision-cut lung slices (PCLSs) from healthy human donors under mild hypercapnic (50–60 mmHg, pH 7.4) or normocapnic conditions for 7 days (Figure 1D). 3D imaging revealed increased smooth muscle thickness and ECM deposition (type I collagen and LTBP2) in hypercapnia-exposed PCLSs, localized to α-SMA–positive regions (Supplemental Videos 1 and 2). Similar responses were observed in human ASMCs, PASMCs, and lung fibroblasts exposed to equivalent hypercapnia in vitro (Supplemental Figure 2, I and J). To assess clinical relevance, we analyzed PCLSs from COPD lungs. COPD-derived slices showed increases in α-SMA thickness, type I collagen, and LTBP2 depositions (Figure 1E and Supplemental Video 3), exceeding the changes observed in hypercapnic donor PCLSs. Histological evaluation of explanted COPD lungs confirmed similar bronchovascular remodeling (Figure 1F).

Together, our findings support a conceptual model in which chronic hypercapnia functions as a persistent microenvironmental stressor promoting lung remodeling. Elevated CO_2_ exposure may alter mesenchymal cell plasticity through changes in gene expression (1), calcium signaling (1), and ECM interactions. In this context, we propose that elevated CO_2_, observed in smokers and individuals with obesity hypoventilation syndrome, may act as an exposome-like factor linking environmental and physiological exposures to structural determinants of airway and vascular pathology.

COPD is a heterogeneous disease characterized by emphysematous destruction, airway remodeling, and vascular changes (3, 4). Our findings implicate hypercapnia specifically in airway and vascular remodeling, even in the absence of emphysematous damage. Importantly, these changes appear to be at least partially reversible upon CO_2_ normalization. Given that noninvasive ventilation improves clinical outcomes in hypercapnic patients (5), our data provide mechanistic support for therapeutic strategies aimed at correcting CO_2_ retention.

## Supplementary Material

Supplemental data

Unedited blot and gel images

Supplemental video 1

Supplemental video 2

Supplemental video 3

Supporting data values

## Figures and Tables

**Figure 1:**
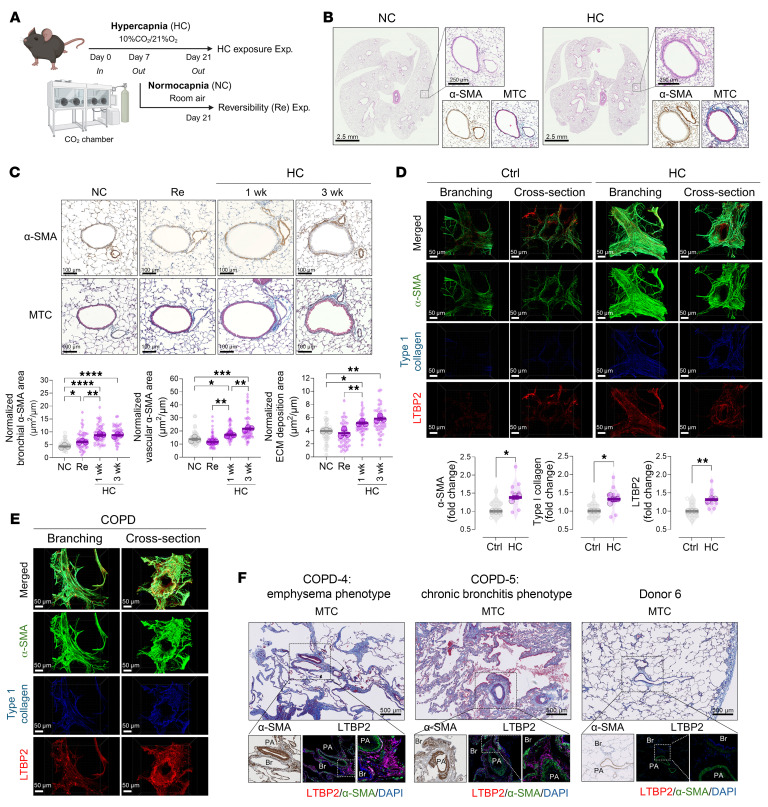
Hypercapnia induces airway and vascular remodeling that reflects COPD histopathology. (**A**–**C**) In vivo studies (*n* = 3 biological replicates). (**A**) Schematic of the experimental design (created with BioRender.com. (**B**) Representative lung sections at day 21. α-SMA, chromogenic IHC. (**C**) Semiquantification of bronchial α-SMA area (*n* = 39–45 airways), pulmonary arterial α-SMA (*n* = 48–52 pulmonary arteries), and ECM deposition (*n* = 39–40 bronchovascular bundles). (**D**–**F**) Human lung tissue. (**D** and **E**) Representative 3D immunofluorescence images and semiquantification of α-SMA, type I collagen, and LTBP2 in PCLSs from (**D**) healthy donors cultured under control (Ctrl) (30–40 mmHg CO_2_, pH 7.4) or buffered hypercapnia (50–60 mmHg CO_2_, pH 7.4) for 7 days, and (**E**) COPD donors (*n* = 8–9 bronchioles from 3 donors). (**F**) Representative images of lung sections from end-stage COPD (*n* = 2) and non-COPD (*n* = 3) donors stained with MTC, α-SMA, or LTBP2. Br, bronchiole; PA, pulmonary artery. Data are presented as a superplot, with individual data points and mean ± SEM values from 3 biological replicates shown. *P < 0.05, **P < 0.01, ***P < 0.001, and ****P < 0.0001, by 1-way ANOVA with Tukey’s post hoc test (**C**) and 2-tailed Welch’s *t* test (**D**). Scale bars: 2.5 mm, 250 μm inset (**B**), 100 μm (**C**), 50 μm (**D** and **E**), and 500 μm (**F**). Exp., experiment; HC, hypercapnia; MTC, Masson’s trichrome; NC, normocapnia; Re, reversibility.

## Version 1 08/26/2025

In-Press Preview

## Version 2 11/03/2025

Electronic publication
