# Supplementary material for: Hypercapnia promotes maladaptive airway and vascular remodeling in mice
Source: J Clin Invest. 2025 Aug 26;135(21):e196928. doi: 10.1172/JCI196928 (PMC12578406; doi:10.1172/JCI196928)
Supplement: Supplemental data [file jci-135-196928-s127.pdf]

Supplemental Materials for:

Hypercapnia promotes maladaptive airway and vascular remodeling in mice

Masahiko Shigemura[1], Felix L. Nuñez Santana[1], S. Marina Casalino-Matsuda[2], David Kirchenbuechler[3], Radmila Nafikova[2], Fei Chen[2], Zhan Yu[2], Yuliana V. Sokolenko[2], Estefani Diaz[4], Suchitra Swaminathan[2], Suror Mohsin[2], Rizaldy P. Scott[4,5], Lynn C. Welch[2], Chitaru Kurihara[1], Emilia Lecuona[1], G.R. Scott Budinger[2,6], Peter H.S. Sporn[2,6], Jacob I. Sznajder[2], and Ankit Bharat[1]

Affiliations: [1]Division of Thoracic Surgery, [2]Division of Pulmonary and Critical Care Medicine, [3]Center for Advanced Microscopy, [4]Robert H. Lurie Comprehensive Cancer Research Center, and [5]Department of Pathology, Feinberg School of Medicine, Northwestern University, Chicago, IL, USA
[6]Research Service, Jesse Brown Veterans Affairs Medical Center, Chicago, IL, USA

Address Correspondence to: Masahiko Shigemura. Division of Thoracic Surgery, Feinberg School of Medicine, Northwestern University, Chicago, IL, USA. Phone: 1.312.503.5033**;** Email: masahiko.shigemura@northwestern.edu

This file contains:

Methods
Authorship contributions
Acknowledgements
Supplementary references
Supplemental Figure 1-3
Supplemental Table 1

Methods

Sex as a biological variable

Our study included both male and female mice and human models. Similar effects of hypercapnia on airway contractility were confirmed in both sexes (1), and thus sex was not considered a biological variable in our analysis.

Animal model and experimental Conditions

We designed an animal study to investigate whether chronic hypercapnia induces structural alterations in the lung. Specifically, we aimed to assess the time course of these changes and their potential reversibility. Four experimental groups were established: (i) normocapnic controls, (ii) 7-day hypercapnia, (iii) 21-day hypercapnia, and (iv) a reversibility group, as detailed below.

Sample size estimates were informed by pilot data, previous experience in our laboratory, and published literature (1-3). Predefined exclusion criteria included sample processing errors identified during the experimental workflow (1). At study completion, each group included three biologically independent replicates. All mice were randomized into experimental groups, and animal care and experimental handling were performed in a blinded and unbiased manner by investigators and technicians.

A total 12 adult C57BL/6J mice (male and female, 10–14 weeks old; Strain #: 000664) were obtained from the Jackson Laboratories (Bar Harbor, ME). Animals were housed under standard conditions with ad libitum access to food and water, a 14-hour light/10-hour dark cycle, and were handled in accordance with National Institutes of Health guidelines. For hypercapnia exposure, mice were placed in a Biospherix C-Shuttle Glove Box (BioSpherix) for up to 21 days. The chamber's atmosphere was monitored twice daily and regulated using ProOx/ProCO_2 controllers (BioSpherix) to maintain a target gas composition of 10% CO_2 and 21% O_2. Temperature was maintained between 20°C and 26°C and relative humidity between 30% and 50% as needed. These settings resulted in arterial partial pressure of carbon dioxide ($PaCO_2$) of ~75 mmHg and PaO_2 of ~100 mmHg (2, 4). In normocapnic control mice maintained in room air, $PaCO_2$ was ~40 mmHg with PaO_2 ~100 mmHg (2, 4). After exposure to normoxic hypercapnia for 3 days, the arterial pH increased, reflecting renal compensation for the respiratory acidosis (2, 4). To assess the reversibility of hypercapnia-induced structural alterations, a subset of mice previously exposed to 7 days of hypercapnia were returned to normocapnic conditions for an additional 14 day-recovery period (Figure 1A). No animals exhibited observable signs of distress during the course of the study. Mice were euthanized with Euthasol (pentobarbital sodium–phenytoin sodium), and whole lungs were collected for subsequent analyses.

Histological assessments in mouse lungs

Lungs were fixed and processed as previously described (5). Briefly, paraffin-embedded lungs were sectioned at 4 μm thickness and stained with H&E, Masson's trichrome (MTC) and chromogenic IHC using antibodies against α-SMA (Cell Signaling, 19245S; 1:1000 dilution) and MCM2 (Cell Signaling, 3619S; 1:800 dilution). An anti-rabbit antibody-HRP polymer conjugate (Biocare Medical, MACH2 and MHRP520) was used in conjunction with the chromogenic substrate DAB to visualize the primary antibody binding sites. IHC slides were counterstained with hematoxylin.

Bright-field images were acquired using a NanoZoomer 2.0 HT scanner (Hamamatsu Photonics K.K.). Morphometric analyses of smooth muscle and extracellular matrix (ECM) deposition were performed using NDP.view2 (Hamamatsu Photonics K.K.) and ImageJ/Fiji software (NIH), respectively.

Smooth muscle area was quantified based on α-SMA immunostaining in airway and pulmonary arterial structures, explicitly excluding pulmonary veins, and was normalized to the perimeter of the airway basement membrane or the luminal circumference of the pulmonary artery for comparative analyses. ECM deposition area was assessed based on MTC-positive staining within each broncho-vascular sheath and normalized to the combined perimeter of airway basement membrane and pulmonary arterial lumens. To ensure consistency, only near-circular cross-sections of airways and vessels were included; sections exhibiting partial collapse were excluded. For each lung section, 11–16 airways, 14–19 pulmonary arteries and 10-17 broncho-vascular bundles were analyzed.

Quantitative data are presented as a superplot, with individual data points and mean values from 3 biological replicates; error bars indicate SEM (Figure 1C).

Cell lines

Human airway smooth muscle cells (PCS-130-011), human pulmonary arterial smooth muscle cell (PCS-100-023), mouse lung fibroblasts (MLg 2908; CCL-206) and human lung fibroblast (PCS-201-013) were obtained from ATCC (Manassas, VA). Cells were maintained in culture medium consisting of DMEM supplemented with 10% FBS, 100 U/mL penicillin, and 100 μg/mL streptomycin. Cultures were incubated at 37°C in a humidified atmosphere containing 5% CO_2.

CO_2 medium and CO_2 exposure

For the different experimental conditions, initial solutions were prepared with DMEM/Ham's F-12 medium/Tris base/MOPS base (3:1:0.25:0.25) containing culture medium, as described elsewhere (1, 2). The buffering capacity of the medium was modified by changing its initial pH with Tris and MOPS base to obtain a pH of 7.4 at the various CO_2 levels (pCO_2 of 5, 8, and 20% for 30-40, 50-60, and ~120 mmHg, respectively). The desired CO_2 and pH levels were achieved by equilibrating

the medium overnight in a humidified chamber (C-Chamber, BioSpherix). The atmosphere of the C-Chamber was controlled with a PRO CO_2 carbon dioxide controller (BioSpherix). In this chamber, cells were exposed to the desired pCO_2 while maintaining 21% O_2 balanced with N_2. Before CO_2 exposure, pH, pCO_2, and pO_2 levels in the medium were measured using a Stat Profile PRIME CCS analyzer (Nova Biomedical).

Cell culture experiments

Cell culture experiments were initiated by replacing the existing medium with CO_2-equilibrated medium, followed by incubation in a C-Chamber under the desired conditions and duration. In previous studies (1), hypercapnic airway smooth muscle cells exhibited maximal hyperreactivity and contractile responses following 2 to 7 days of exposure to high CO_2 (~120 mmHg, extracellular pH 7.4). Accordingly, several in vitro experiments were conducted under 3-day high CO_2 exposure conditions (~120 mmHg, extracellular pH 7.4). Notably, lower levels of CO_2 are also capable of activating relevant signaling pathways and eliciting pathophysiologic effects, although typically requiring more prolonged exposure durations (1).

Cell lysate and Western blot analysis

Cells were homogenized in lysis buffer (Cell Signaling). The protein concentration was quantified by the Bradford assay (Bio-Rad), and proteins were resolved on a 4-20% gradient polyacrylamide gels (Bio-Rad). Thereafter, proteins were transferred to nitrocellulose membranes (Bio-Rad) using a Trans-Blot Turbo transfer system (Bio-Rad). Incubation with anti-α-SMA antibody (R&D systems, MAB1420; 1:2000 dilution) was performed overnight at 4°C. Blots were developed with a chemiluminescence detection kit (SuperSignal™ West Femto Maximum Sensitivity Substrate, ThermoFisher) as recommended by the manufacturer. The bands were scanned and quantified using a LI-COR Fc Odyssey Imaging system and companion software Image Studio version 5.2 (LI-COR Biosciences). Anti-β-Tubulin (Cell Signaling, 2146S; 1:200 dilution) was used as a loading control.

F-actin staining of the cell and confocal imaging

Cells were fixed on glass coverslips with 3.7% formaldehyde for 10 min, washed three times with PBS for 10 min and stained with Alexa Fluor 488-conjugated phalloidin (Invitrogen, A12379; 1:500) containing DAPI (Sigma-Aldrich) in PBS for 15 min. After washing cells three times with PBS for 10 min, they were mounted on glass slides with Fluoromount G (Southern Biotech) and imaged using a Zeiss Axio Imager Z2 with ApoTome.2 microscope equipped with Axiocam 503 Mono, X-Cite 120 LED Boost System, and Zen 2.3 software (Carl Zeiss). Single-cell images were analyzed using ImageJ/Fiji software (NIH) for the integrated fluorescence intensity. The fluorescence intensity was averaged to calculate the integrated fluorescent intensity from each slice in the single cell.

Averaged values from all 8 slices in each single cell stack were then averaged to collect the cell's integrated fluorescent intensity. This intensity was normalized to the cell area for plotting and comparative analyses.

Quantitative reverse transcription PCR

Total RNA was extracted using the RNeasy plus kit (Qiagen). cDNA was synthesized from 200 ng - 1 µg of total RNA using an iScript cDNA Synthesis kit (Bio-rad) and mRNA expression level was determined by quantitative PCR (qPCR) using the SsoAdvanced Universal Inhibitor-Tolerant SYBR Green Supermix (Bio-Rad). Relative expression of the transcripts was determined according to the $\Delta\Delta C_t$ method using *Rpl19* for MLg 2908, and *RPL19* for hASMC, hPASMC and hLF as reference for normalization.

The following primers were used for mouse samples: *Rpl19*-F, 5'-GAAATCGCCAATGCCAACTC-3'; *Rpl19*-R, 5'- CTTCAGGTACAGGCTGTGATAC-3'; *Col1a1*-F, 5'-GCTTGAAGACCTATGTGGGTATAA-3'; *Col1a1*-R, 5'-GGTGGAGAAAGGAGCAGAAA-3'; *Ltbp2*-F, 5'-CCCTGCTCTCAAATCCTGG-3'; *Ltbp2*-R, 5'-GGGATGTAACCTTGACCACTG-3'.

The following primers were used for human samples: *RPL19*-F, 5'- ATGCCAGAGAAGGTCACATG -3'; *RPL19*-R, 5'-ACACATTCCCCTTCACCTTC-3'; *COL1A1*-F, 5'-CCCCTGGAAAGAATGGAGATG-3'; *COL1A1*-R, 5'-TCCAAACCACTGAAACCTCTG-3'; *LTBP2*-F, 5'-AGGAAAGGACACTGCCAAG-3'; *LTBP2*-R, 5'-TCATTCACATCTACACAGCTCC-3'.

Secondary analysis of available transcriptomic datasets of hypercapnia-exposed fibroblasts

To explore hypercapnia-responsive ECM genes in lung fibroblasts, secondary transcriptomic analysis was performed using the dataset from a study (3) investigating the transcriptomic response to hypercapnia in PDGFRα-expressing fibroblasts isolated from C57BL/6J mice exposed to normoxic hypercapnia (10%CO_2, 21%O_2) for 10 days (GSE193538). Significant differentially expressed genes (DEGs) were determined as any genes with ≥ 0.49 $\log_2$ fold-change with an adjusted p value ≤ 0.05. Hypercapnia-responsive genes in the dataset were compared with the Lung Gene Expression in Single-Cell (LungGENS) program (6, 7) which can distinguish the genes of mesenchymal subtypes into those of myofibroblast, intermediate fibroblast and matrix fibroblast. We identified *Ltbp2* as a hypercapnia-responsive ECM gene with high fold-change expression. LTBP2 has been reported to play a complex and context-dependent role in elastic fiber assembly. While early findings suggested a promoting effect on elastogenesis (8), more detailed mechanistic investigations have shown that LTBP2 may inhibit key interactions necessary for efficient elastic fiber formation (9). Our preliminary data showed that hypercapnia did not alter elastin abundance or lung elastance (Supplemental Figure 3), suggesting that the contribution of LTBP2 to

elastogenesis under hypercapnic conditions may be limited. Recent studies have proposed that LTBP2 may play a role in regulating myofibroblast differentiation (10-12).

Human Precision Cut Lung Slices (hPCLS)

Human PCLS from healthy and COPD donors were purchased from AnaBios Corporation which recovers human lung via the U.S.-based organ procurement network. The clinical summaries of anonymized donors, including information on respiratory conditions and the presence of COPD, were provided by AnaBios. These summaries were compiled from data provided by organ procurement centers. One PCLS sample was obtained from lung biopsies of a healthy organ donor at Northwestern Medicine, following standard perfusion protocols prior to transplantation (13) at Northwestern Medicine. Biopsy samples were infused with a 2.5% (w/v) low melting agarose (Promega) in HBSS, then sectioned into 350-μm slices using a VT1200S Leica vibratome set to 3.0-mm amplitude and 0.7 mm/s speed, as described previously (14). Lung slices were incubated at 37°C in culture medium. The medium was replaced hourly for the first three hours. For cryopreservation, PCLS were stored in 10% DMSO diluted in DMEM/F-12 medium in liquid nitrogen, following previously established protocol (15).

On the day prior to the experiment, PCLS were rapidly thawed in a 37°C water bath, carefully washed once, and incubated overnight in DMEM/F-12 medium. Subsequently, the medium was replaced with CO_2-equilibrated medium, and PCLS were incubated in the C-Chamber for 7 days. The buffering capacity of the CO_2-equilibrated medium was modified by changing its initial pH with Tris and MOPS base to obtain a pH of 7.4 at the various CO_2 levels as described above.

The study population characteristics, including age, ethnicity, gender, BMI, cause of death (COD) are shown in Supplemental Table 1.

Histological assessments in hPCLS

Human PCLS were fixed in 10% formalin for 10 min, permeabilized with 0.5% Triton X-100 in PBS for 5 min and washed six times with PBS for 1 hour. The PCLS were blocked with 10% normal goat serum in PBS for 1 hour at room temperature and incubated overnight at 4°C with primary antibodies: α-SMA (R&D systems, MAB1420; 1:500 dilution), LTBP2 (Proteintec, 17708-1-AP; 1:500 dilution) and collagen type I (Novus Biologicals, NBP600-408AF350; 1:400 dilution) in 10% normal goat serum. Lung slices were washed three times with PBS for 10 min and incubated with secondary antibodies conjugated to fluorescein isothiocyanate (Invitrogen, A-11017 and A-31573; 1:1000 dilution) in 10% normal goat serum for 2 hours at room temperature. PCLS were washed three times with PBS and stored in 12-well plates at 4°C until use.

To preserve and visualize the 3D structure of the bronchial airways and surrounding tissue without compression, immunostained PCLS were mounted on a glass slide using a SecureSeal™ silicone

spacer (#70336-10, Electron Microscopy Science) filled with 350 µl of PBS and covered with a coverslip. The mounted slices were imaged using a Leica SP8 Dive Multiphoton system with 2 Spectra Physics lasers: Mai Tai DeepSee (690-1040 nm) tuned at 840 nm and a Spectra Physics InSight X3 (680-1300nm) tuned at 1200 nm. We used a 25X magnification objective (HC PL IRAPO 25x/1.00 Water) equipped with a motorized correction collar. A volume of 500 × 500 × 150 µm with a pixel size of xy and a step size of 1 µm were obtained for each PCLS.

3D fluorescence images of PCLS were semi-quantitatively analyzed with the IMARIS 10.0.0 software (Bitplane). The Volume of the PCLS were determined with the surface function where a threshold for α-SMA (Filter: 492nm - 545nm) was applied. The same thresholds were used for all datasets. The Volume and the Intensities of type I collagen (Filter: 424nm - 463nm) and LTBP-2 (Filter: 642nm - 695nm) were measured and the density (α-SMA) of intensity inside the Volume was calculated. The ratio of density of α-SMA to I collagen and LTBP-2 were calculated. For comparative analysis, 2–3 different shapes of bronchioles (e.g., straight, branching, or cross-sectional) were selected from each PCLS, ensuring similar structure and volume between experimental groups. Within these 3D bronchiolar regions, all smooth muscle structures including adjacent pulmonary arteries and veins were visualized based on α-SMA labeling. Accordingly, we semi-quantified total smooth muscle content (bronchiolar and pulmonary vascular) and associated ECM deposition.

Representative 3D projections of healthy PCLS cultured under control or buffered hypercapnia conditions, and of COPD PCLS, are shown in Supplemental Videos 1-3. Quantitative data are presented as a superplot, with individual data points and mean values from 3 biological replicates; error bars indicate SEM (Figure 1D).

Histological assessments in human lung biopsies

Lung biopsies were obtained from donor lung allograft treated by standard perfusion protocols prior to implantation (13) or from COPD-explanted lungs at Northwestern Medicine. The study population, including age, ethnicity, gender, body mass index, cause of death and COPD phenotype, are shown in Supplemental Table 1.

The biopsies were fixed, processed and embedded, and sectioned into 4-µm slices, which were stained with H&E, MTC, chromogenic IHC with anti-α-SMA (Cell Signaling, 19245S; 1:1000 dilution) antibody as described below, and immunofluorescence with anti-α-SMA (R&D systems, MAB1420; 1:500 dilution) and -LTBP2 (Proteintec, 17708-1-AP; 1:500 dilution) antibodies. Bright-field images were acquired using a NanoZoomer 2.0 HT scanner (Hamamatsu Photonics K.K.). Immunofluorescence images were captured using a Zeiss Axio Imager Z2 with ApoTome.3 microscope equipped with an Axiocam 503 Mono, X-Cite 120 LED Boost System, and Zen 2.3 software (Carl Zeiss).

Statistical analysis

Statistical methods are described in the figure legends and in relevant method descriptions. Sample size (n) used for statistical analyses are provided in the relevant figures. Statistical analyses were performed using Prism (version 10.4.1, GraphPad Software). Unpaired Student's *t*-tests were applied for comparisons assuming equal variances, while Welch's *t*-tests were used when variances differed. Ordinary one-way ANOVA was used to compare means across multiple groups with equal variances, followed by Tukey's post-hoc test for multiple comparisons. Variances were assessed by *F*-test or the Brown-Forsythe test. For the qPCR data analysis, we performed one-sample *t*-test against a hypothetical mean of 1 in the control group. Statistical outliers were identified and removed based on Grubbs' test criteria when appropriate. A p-value <0.05 was considered statistically significant. Unless otherwise stated, data are presented as means ± S.E.M.

Study approval

All procedures involving animals were approved by the Northwestern University Institutional Animal Care and Use Committee (IACUC; Protocols IS00010662 and IS00004576). The human research protocol was approved by the Northwestern University Institutional Review Board (STU00219570, STU00212120). All study subjects were informed and provided written consent prior to study.

Data availability

Data of the findings of this study are included in the main article and supplementary material (see Supporting Data Values file). Further information is available from the corresponding authors upon request.

Author contributions

M.S. conceived and designed the study, performed experiments, interpreted the data, and wrote the manuscript. F.L.N.S., D.K., R.N., and R.P.S. conducted microscopy experiments and contributed to data analysis. Z.Y., Y.V.S., E.D., S.S., S.M., and C.K. collected human lung biopsy specimens. F.C. and L.C.M. carried out the animal experiments. E.L. and S.M.C contributed to data interpretation and manuscript writing. G.R.S.B., P.H.S.S., J.I.S., and A.B. interpreted the data, contributed to manuscript writing, and provided funding and resources.

Acknowledgments

We thank Akshay Govindan for secondary RNA-seq analysis, Dr. Alexander Misharin for assistance with human lung biopsy collection, and Haiying Sun and Dr. Yudai Miyashita for their support and insightful discussions. Comparative histopathology and molecular phenotyping were

performed by the Mouse Histology and Phenotyping Laboratory (MHPL, RRID:SCR_017870) and the Pathology Core Facility (PATHCORE) at Northwestern University, both supported by NCI grant 5P30CA060553-30. This work was supported in whole or in part by the Department of Veterans Affairs (I01CX002350) and the NIH (HL147070, HL173987, HL131745, HL145478, HL147290, HL147575, HL173940, and P01HL169188) and is subject to the NIH Public Access Policy. Through acceptance of this federal funding, the NIH has been granted the right to make the work publicly available in PubMed Central.

Supplementary references

1. Shigemura M, Lecuona E, Angulo M, Homma T, Rodriguez DA, Gonzalez-Gonzalez FJ, et al. Hypercapnia increases airway smooth muscle contractility via caspase-7-mediated miR-133a-RhoA signaling. *Sci Transl Med.* 2018;10(457).

2. Jaitovich A, Angulo M, Lecuona E, Dada LA, Welch LC, Cheng Y, et al. High CO2 levels cause skeletal muscle atrophy via AMP-activated kinase (AMPK), FoxO3a protein, and muscle-specific Ring finger protein 1 (MuRF1). *J Biol Chem.* 2015;290(14):9183-94.

3. Dada LA, Welch LC, Magnani ND, Ren Z, Han H, Brazee PL, et al. Hypercapnia alters stroma-derived Wnt production to limit beta-catenin signaling and proliferation in AT2 cells. *JCI Insight.* 2023;8(4).

4. Gates KL, Howell HA, Nair A, Vohwinkel CU, Welch LC, Beitel GJ, et al. Hypercapnia impairs lung neutrophil function and increases mortality in murine pseudomonas pneumonia. *Am J Respir Cell Mol Biol.* 2013;49(5):821-8.

5. Magnani ND, Dada LA, Queisser MA, Brazee PL, Welch LC, Anekalla KR, et al. HIF and HOIL-1L-mediated PKCzeta degradation stabilizes plasma membrane Na,K-ATPase to protect against hypoxia-induced lung injury. *Proc Natl Acad Sci U S A.* 2017;114(47):E10178-E86.

6. Du Y, Guo M, Whitsett JA, and Xu Y. 'LungGENS': a web-based tool for mapping single-cell gene expression in the developing lung. *Thorax.* 2015;70(11):1092-4.

7. Du Y, Kitzmiller JA, Sridharan A, Perl AK, Bridges JP, Misra RS, et al. Lung Gene Expression Analysis (LGEA): an integrative web portal for comprehensive gene expression data analysis in lung development. *Thorax.* 2017;72(5):481-4.

8. Hirai M, Horiguchi M, Ohbayashi T, Kita T, Chien KR, and Nakamura T. Latent TGF-beta-binding protein 2 binds to DANCE/fibulin-5 and regulates elastic fiber assembly. *EMBO J.* 2007;26(14):3283-95.

9. Sideek MA, Menz C, Parsi MK, and Gibson MA. LTBP-2 competes with tropoelastin for binding to fibulin-5 and heparin, and is a negative modulator of elastinogenesis. *Matrix Biol.* 2014;34:114-23.

10. Enomoto Y, Matsushima S, Shibata K, Aoshima Y, Yagi H, Meguro S, et al. LTBP2 is secreted from lung myofibroblasts and is a potential biomarker for idiopathic pulmonary fibrosis. *Clin Sci (Lond).* 2018;132(14):1565-80.

11. Zou M, Zou J, Hu X, Zheng W, Zhang M, and Cheng Z. Latent Transforming Growth Factor-beta Binding Protein-2 Regulates Lung Fibroblast-to-Myofibroblast Differentiation in Pulmonary Fibrosis via NF-kappaB Signaling. *Front Pharmacol.* 2021;12:788714.

12. Lu M, Tao S, Zhao C, Wang N, Hu Q, Li Q, et al. HIF-1alpha/LTBP2 axis activate HSCs to promote liver fibrosis by interacting with LOXL1 via the ERK pathway. *Cellular and molecular life sciences : CMLS.* 2025;82(1):161.

13. Zheng Z, Chiu S, Akbarpour M, Sun H, Reyfman PA, Anekalla KR, et al. Donor pulmonary intravascular nonclassical monocytes recruit recipient neutrophils and mediate primary lung allograft dysfunction. *Sci Transl Med.* 2017;9(394).

14. Bharat A, Querrey M, Markov NS, Kim S, Kurihara C, Garza-Castillon R, et al. Lung transplantation for patients with severe COVID-19. *Sci Transl Med.* 2020;12(574).

15. Rosner SR, Ram-Mohan S, Paez-Cortez JR, Lavoie TL, Dowell ML, Yuan L, et al. Airway contractility in the precision-cut lung slice after cryopreservation. *Am J Respir Cell Mol Biol.* 2014;50(5):876-81.

Supplemental figures and table

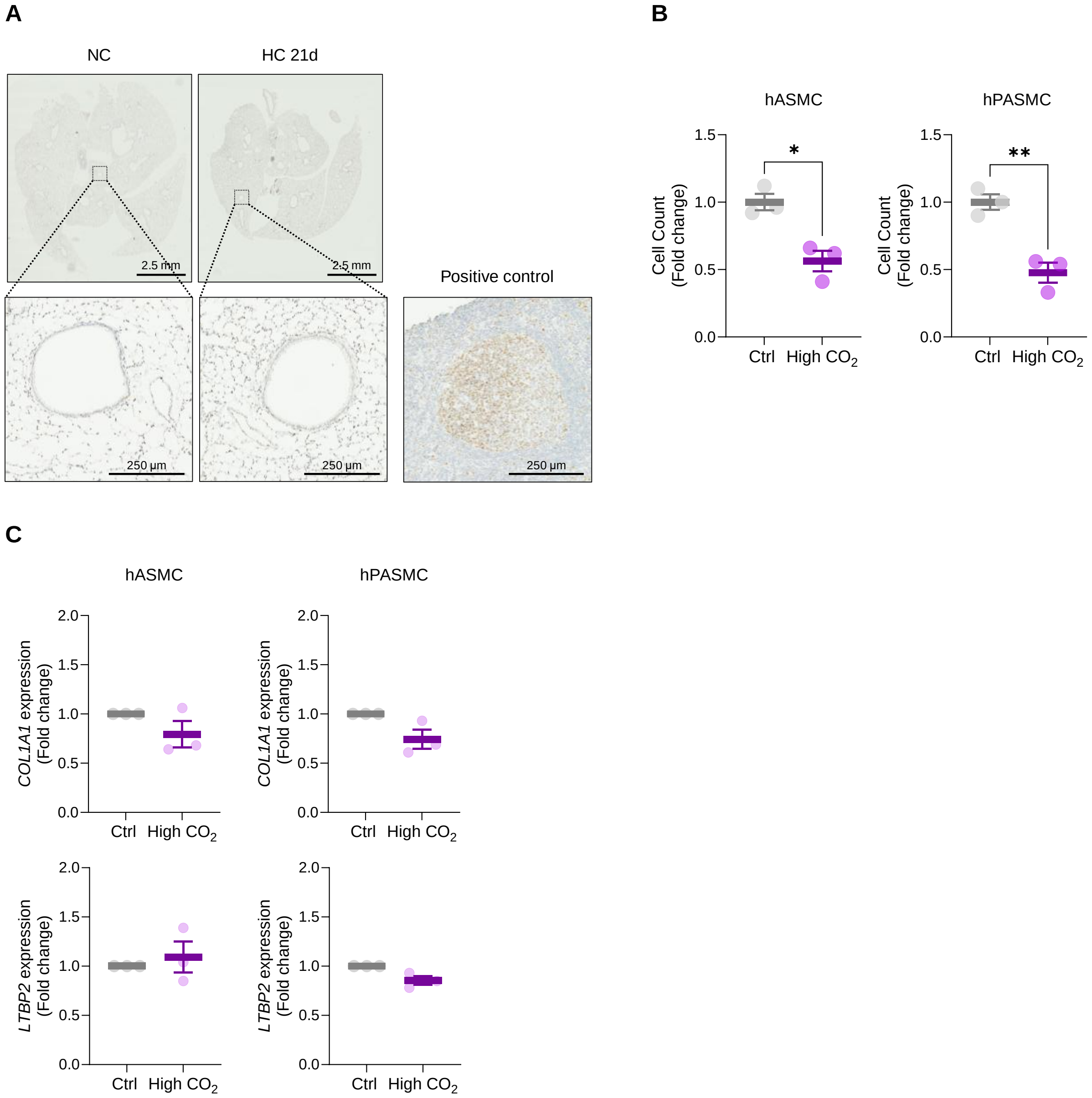

Supplemental Figure 1. ***Hypercapnia does not increase cell proliferation and ECM gene expression in lung smooth muscle cells.***

(**A**) Representative images of chromogenic immunohistochemical staining of minichromosome maintenance complex component 2 (MCM2) in mouse lung tissues exposed to room air (normocapnia, NC) or normoxic hypercapnia (HC: 10%CO_2 and 21%O_2) for 21 days (n= 3 mice). Positive control is human tonsil. (**B-C**) Human airway smooth muscle cells (hASMC) and

pulmonary arterial smooth muscle cells (hPASMC) were cultured under control (Ctrl: 30-40 mmHg CO_2, pH 7.4) or high CO_2 (~120 mmHg CO_2, pH 7.4) for 3 days (n=3 independent replicates). (**B**) The number of cells. (**C**) *COL1A1* and *LTBP2* expression.

Data represent mean ± SEM. Statistical significance was determined by unpaired *t*-test (**B**) and one-sample *t*-test (**C**). *p<0.05, **p<0.01.

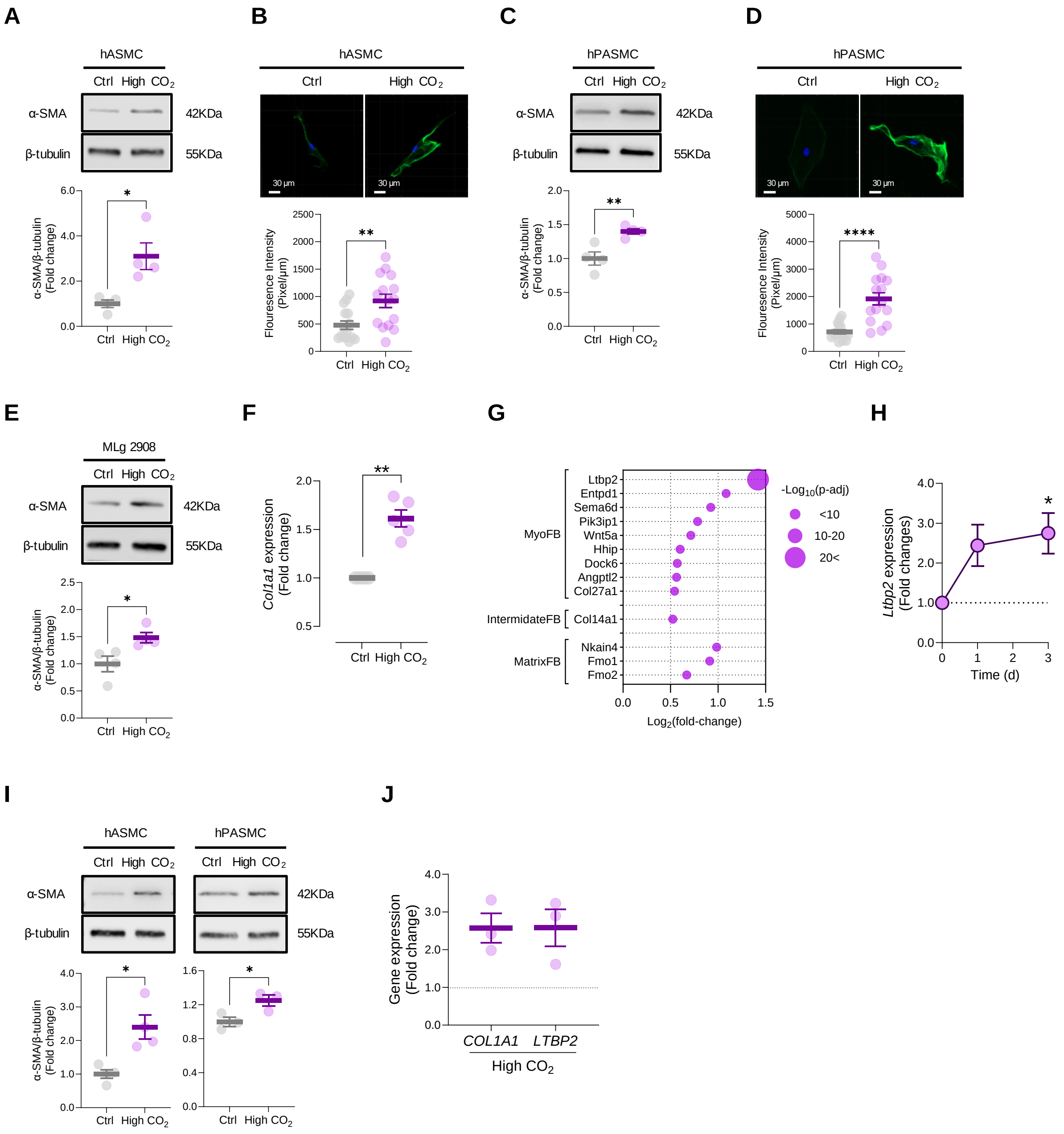

Supplemental Figure 2. ***Hypercapnia induces phenotypic shifts toward a contractile smooth muscle and myofibroblast-like phenotype.***

(**A**-**H**) Cells were cultured under control (Ctrl: 30-40 mmHg CO_2, pH 7.4) or high CO_2 (~120 mmHg CO_2, pH 7.4) for 3 days (n=3-5 independent replicates). (**A, C, E**) Western Blot and quantification of α-SMA in human ASMC (hASMC) and PASMC (hPASMC), and mouse lung fibroblast (MLg2908). (**B, D**) Phalloidin F-actin staining and quantification in hASMC and hPASMC (n=15 cells). (**F**) *Col1a1* expression in MLg2908. (**G**) Transcriptomic profiling of hypercapnia-exposed Pdgfra^{+} fibroblasts (GSE164733). Differentially expressed genes (DEGs) categorized with fibroblast (FB) subtypes. (**H**)

Ltbp2 expression in MLg2908. (**I**-**J**) hASMC, hPASMC and human lung fibroblasts (hLF) were cultured under Ctrl or high CO_2 (50-60 mmHg CO_2, pH 7.4) for 7 days. (**I**) α-SMA expression in hASMC and hPASMC (n=3-4 independent replicates). (**J**) *COL1A1* and *LTBP2* expression in hLF (n=3-4 independent replicates).

Data represent mean ± SEM. Statistical significance was determined by unpaired *t*-test (**A-C**, **E**, **I**), Welch's *t*-test (**D**) and one-sample *t*-test (**F, H**). *p<0.05, **p<0.01, ****p<0.0001.

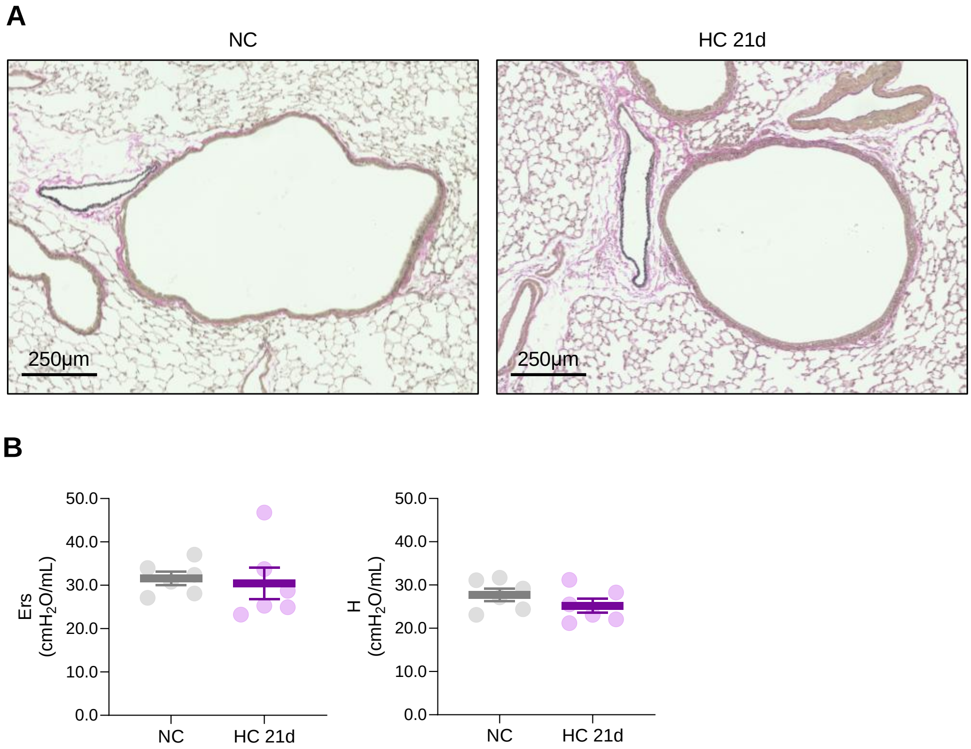

Supplemental Figure 3. ***Hypercapnia does not alter elastin abundance or lung elastance.***

C57BL/6J mice were exposed to room air (normocapnia, NC) or normoxic hypercapnia (HC: 10%CO_2 and 21%O_2) for 21 days. (**A**) Representative images of Verhoeff-Van Gieson staining (n=3 mice). Elastin, Black/dark purple; collagen, pink; smooth muscle and other structures, yellow/orange. (**B**) Respiratory system elastance (Ers, left) and tissue elastance (H, right) measured using the flexiVent system (n=6 mice). A secondary analysis was performed using a previously published dataset (1). Data represent mean ± SEM. Statistical significance was determined by unpaired *t*-test.

Supplemental Table 1. ***Demographic characteristics of study subjects.***

Human PCLS research

Subject	Age (yr)	Ethnicity	Gender	BMI (kg/m^2)	COD
Donor-1	51	White	Female	20.3	CVA/ICH/Stroke
Donor-2	35	White	Female	19.5	Anoxia
Donor-3	17	White	Male	20.0	Anoxia
COPD-1	48	White	Female	29.0	Anoxia
COPD-2	39	White	Male	30.0	Anoxia
COPD-3	48	Hispanic/Latino	Male	25.0	Head trauma

Histological evaluation of explanted COPD lungs

Subject	Age (yr)	Ethnicity	Gender	BMI (kg/m^2)	COD[A]	COPD phenotype
Donor-4	18	African American	Male	21.5	Anoxia	N/A[B]
Donor-5	30	White	Female	31.2	Anoxia	N/A[B]
Donor-6	29	African American	Male	27.1	Head trauma	N/A[B]
COPD-4	64	White	Male	26.4	N/A	Emphysema
COPD-5	69	White	Female	21.9	N/A	Chronic bronchitis

[A] COD, Cause of death; [B] N/A, not applicable.
