# Supplementary material for: Hypercapnia promotes maladaptive airway and vascular remodeling in mice
Source: J Clin Invest. 2025 Aug 26;135(21):e196928. doi: 10.1172/JCI196928 (PMC12578406; doi:10.1172/JCI196928)
Supplement: Unedited blot and gel images [file jci-135-196928-s128.pdf]

Unedited blot and gel images

For Supplemental Figure 2A:

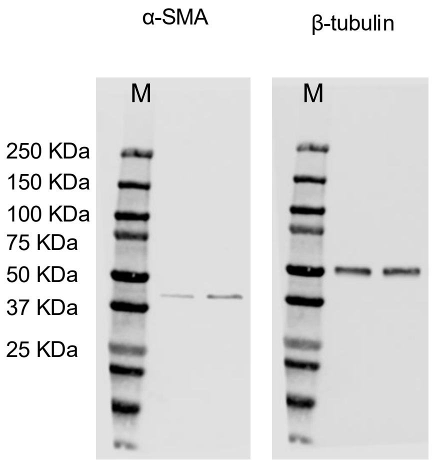

M, protein marker

For Supplemental Figure 2C:

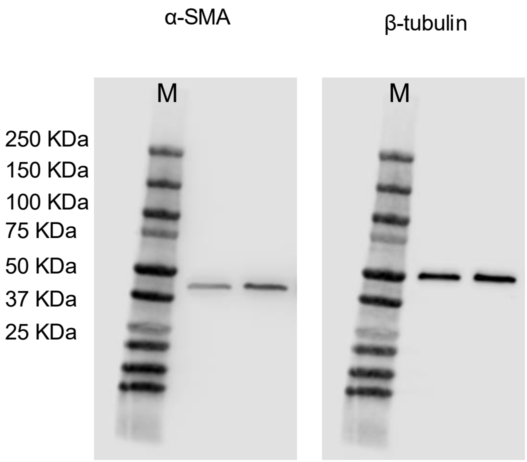

M, protein marker

For Supplemental Figure 2E:

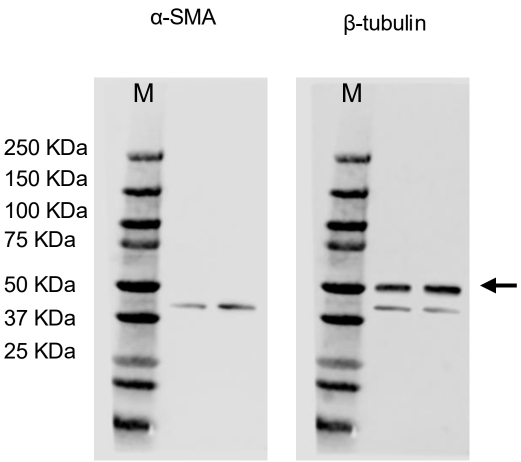

M, protein marker

For Supplemental Figure 2I:

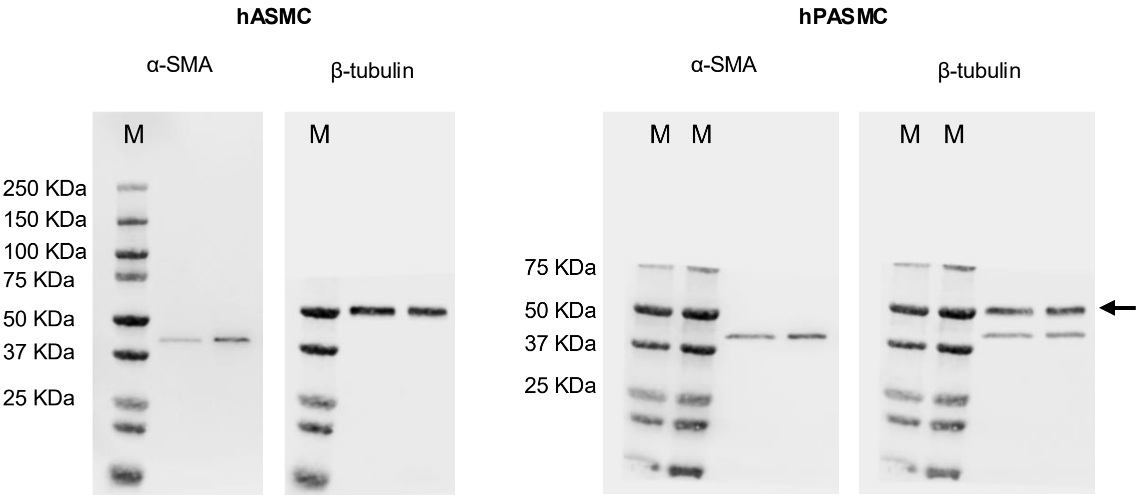

M, protein marker
